# Laparotomy for Fibro-Cystic Tumor

**Published:** 1893-01

**Authors:** R. E. Houghton

**Affiliations:** Midland, Texas


For Daniel's Texas Medical Journal.

### LAPAROTOMY FOR FIBRO CYSTIC TUMOR.

BY R. E. HAUGHTON, M. D.

[Read before the Northwest Texas Medical Association at Cisco, on Tuesday, Dec. 6th, 1892.]

CASE: Mrs. B., age 37 years, consulted me about a tumor with a view to diagnosis and removal. It was believed to be by her physician and friends, an ovarian tumor. She was anxious to have it removed, if possible, as her health was impairing and already much impaired, and the size of the growth had reached that of a full term pregnancy. I examined all the means used to determine diagnosis, and decided that it presented the conditions of a multilocular ovarian tumor, was clearly a fluctuating tumor, but had deviated from the ordinary history of an ovarian tumor, in this, viz: that it developed centrally while the most common mode, if ovarian, are developed from the right or left ovarian region, unless double, which is not an usual occurrence. This led to the most careful investigation as the suspicion of a fibroid, or fibro cystic tumor growing out from the uterus. She had not menstruated for more than a year, the reason of which will appear in the sequel. This led to a careful examination of the possible condition and relation of the uterus, and it could not be found by any exploration adopted. The conception of the condition was that it had been lifted up from the pelvis, beyond the means of examination, and so it was found to be. In view of her impaired health, and recently more rapid growth, an operation seemed to be the only thing remaining to be done which could give any hope of improvement of her condition, keeping in view the gravity of the risks to be taken in such a procedure. They decided to carefully consider the matter, and if they decided to submit the case to an operation, to notify me and fix a day. I went to the home of the lady, and, in conference with her physician and others, proceeded to the operation. The lady was placed upon a table and chloroform, ether and alcohol used as the anæsthetic. The operation was by the long incision, after a short exploratory one for observation. There was found almost universal adhesions to the other viscera and walls of the abdomen. There was found a poly-cystic tumor arising from the lower portion of the body and neck of the uterus, about the junction of the neck and body of the organ, and by its large development, enclosing the body of the uterus so completely that but a trace of it was left, being compressed and atrophied, so that the canal of the neck and outline of body and thickness of wall was from an eighth to a quarter of an inch only remaining. The adhesions gave rise to some troublesome hemorrhage, which was carefully controlled by ligation and styptics. The broad ligaments and fallopian tubes and ovaries were removed. The broad ligaments being reflexions of the peritoneum, were ligated, in three sections, and attachments separated. One difficulty occurs in such cases, viz: properly constricting the ligated tissues, as elasticity of the tissue plays an important part in effectively controlling the hemorrhage. Lifting up the entire mass and getting behind it and below the junction of the vagina, a strong ligature was cast so as to embrace the entire tumor at the line of junction above the vagina and encircling the atrophied cervix. This is in accord with the suggestion of Schroeder in cases of cancer of the uterus, viz: division between the external and internal os, which he calls the supra-vaginal incision, and which he has made. Not being willing to risk the danger from hemorrhage if a ligature should slip or not be sufficiently tight, the *ecraseur* was used to control the possible hemorrhage, while the mass of tumors, uterus, ovaries and tubes were removed through a ligation of the broad ligaments and the supra-vaginal ligation and excision of the neck of the uterus. Where there was found bleeding or oozing from vessels or abraded surfaces, a solution of the sub. sul. ferri was applied, as it is innocuous to the tissues, which promptly controlled any after loss of blood till the entire cavity was dry, and pelvis and abdomen carefully cleansed with hot water. The patient was removed to bed. She rallied from the shock of the operation, which was for a time profound, yet warm bottles and stimulants soon restored her to a comparatively comfortable condition, and so continued until the morning of the second day. The external wound was closed by several interrupted sutures of silver wire and strips, between the sutures, completely closing the wound, except a point for a drainage tube and ligatures within. The dressing was completed by antiseptic compress and bandage.

She rested well the first night and into the second day, when there was evidence of peritoneal inflammation in fever, pulse increased, tenderness over abdomen; the pulse failed, the temperature higher, growing paleness of surface, pinched features, coldness, and death on the third day.

---

DISCUSSION OF RESULTS.

*History of Fibro Cysts.*—Whether the best thing was done in this case and other such cases by operation can only be determined by a careful study and results obtained. Hence, it is important that medical men report their *failures* as well as their successes. This is the reason why this case appears here to-day, and, if in anything the next operation can be made a success, involving such conditions as were presented here, I shall have been paid. I have made such operations before and since, in which the abdomen was laid open, and every case, so far, has its own peculiar features which must guide the surgeon. I am aware that the technique of the operation, under our antiseptic methods, has vastly improved, yet a review of fatal cases honestly reported will, no doubt, still aid us to secure better results and also enable us to still more improve our methods of diagnosis. Baker Brown says: "I know of no distinguishing signs between utero-fibro cyst and ovarian cystoma. These have long been recognized as a distinct class of tumors, and were first described by Cruveilheir and have not unfrequently been mistaken by the ovariotomist for ovarian cysts. Where the growth has been rapid and fluctuation becoming very evident, an error in diagnosis may readily occur. Hence, to make a more careful and certain diagnosis, an exploratory incision, in my judgment, should be made; yet, even with the finger or hand in the abdominal cavity, it is difficult to distinguish a fibro-cyst from an ovarian cyst. Koberle thinks "that the differential diagnosis ought to be clearly made," and Wells says "that a darker and less pearly blue aspect of the tumor would be sufficient to put the surgeon on his guard against mistaking it for an ovarian cyst. Dr. Atlee admits the difficulty of diagnosis and says that errors are frequent, even in hard fibroid tumors, but much more common in fibro-cystic tumors. He says further: "I believe that a positive diagnosis can be made only by tapping" and testing the fluid by heat (or heat and acid), which is a sufficiently reliable test, but one which is more so is "a fluid coagulable on exposure to air."

We find, so far, medical men have not been and and are not able to make a differential diagnosis unless by this method. The question arises, if I had been able to identify the character of this mass of tumors, as I had fear that it was such as was found, would it have been just and right to subject the woman to a dangerous operation in the hope of saving her? Tapping is now regarded and has often proved a dangerous and fatal method and I did not use this method. Where there is great doubt as to the peculiar nature of the tumor, it may be best to give the patient the benefit of the doubt, and not operate if the diagnosis cannot be cleared up before, yet this leaves the patient to succumb to her fate without doing anything to mitigate her condition. Hope is the anchor of the soul. I report this case, first, because the conditions required a removal of the uterus; secondly, because the nature of the tumor, viz: a fibro-polycystic mass of tumors of various sizes, from that of a large sized child's head of six months old to that of the size of a man's fist, the mass weighing *forty-five pounds*. Some of these tumors contained fluid of a yellow serous kind,—some thick like mel. or honey (melicerous), and some of them were solid or fibroid tumors. They were arranged around the uterus as a centre, springing out from it at the junction of neck and body, similar to a bunch of grapes about the stem, or similar to a bunch of hyatids, which I once obtained in a case of hemorrhage from the uterus, and of which they were the producing cause; thirdly and lastly, because, unfortunately, it was a fatal case, which are not always reported. This case presents several conditions unique and interesting, especially the fact, that the uterus of a child-bearing woman being so compressed and atrophied as to present but a trace of outline, having its functions completely arrested—abolished, and with it the abolition of the function of the ovaries, as ovalation had not existed, by any evidence of menstruation, for months during its progress.

"Dr. Coffin, of New Orleans, Feb., 1861, removed the uterus entire with one ovary and fallopian tube, leaving the other, the woman recovering, being able to sit up on the third day."—*New Orleans Medical News and Hospital Gazette.*

In the *American Journal of Obstetrics*, by Munde, January, 1879, is described a case, given by Freund, of Breslau, with a plate illustrative, and which he calls a new method, which is an operation for complete removal of the uterus, in which the chief consideration is the prevention of hemorrhage, and closure of the peritoneal wound. He does this by uniting the lower portion of both free peritoneal borders with the corresponding lateral portion of the abdominal wound, thereby preventing the detachment of the anterior pelvic attachment of the peritoneum. These operations were for cancer of the uterus, of which he has now performed fourteen operations, of this number eight deaths have been published and five recoveries, and one incomplete operation. Twenty-five operations,—more results known,—19 died, 5 recovered, one incomplete.

I have alluded to this modification, proposed by Schroeder in Freund's operation, as made for cancer of the body of the organ and involving the neck or mouth, so as to secure an operation which may keep clear of cancerous tissue, and by this means avoid the possible complication preventing a final recovery. In such cases as here alluded to the supra-vaginal incision is the only one which should be adopted, if by that means the whole growth can be removed.

One case is reported by Trenholme, where he used the ecraseur, and attributes the fatal result to it, but whether it is better to use it and prevent hemorrhage until a safe ligation can be accomplished with an antiseptic ligature, or allow bleeding, which might destroy the patient suddenly, and prevent a completion of the operation successfully. These are questions which must be met and determined in the mind ot the surgeon, together with such accidents or complications as may occur to any operator.

HYSTEROTOMY—LEGITIMACY OF.—As late as 1875, Schroeder wrote thus: "At all events, it must for the present be left wholly undecided, whether, and how far, the extirpation of the uterus, by laparotomy, may be justifiable. Such a procedure is rational where the life of the patient is jeopardized." And to my mind this is true in any form of disease (aside from cancer), if the patient's life is certainly to be lost, unless an operation can save her. Hence (fibroid, or recurrent fibroids,) polycystic tumors, sarcoma, cancer, and some other forms of disease, are to be carefully investigated, and when, in the judment of the surgeon, he ought to operate, it is only a question to be adjudicated at the bar of conscience. I am sure, in view of the great advance surgery has made on this line with all the means of antiseptics, and cleanliness, and instrumental aids, together with the small per cent. of mortality, developed on various lines, by laparotomy, we should not lose sight of the value of a human life. Perhaps no question will be raised, in view of the number of cases reported as successful. Hegar and Kaltenbach report 89 cases of completed operations; 26 recovered. In a total of 218 cases, 81 recovered; 137 deaths. The mortality is 62.84 per cent. in this operation. Pean, of Paris, had 32 operations and 26 recoveries, and in his work upon hysterotomy for fibroids and fibro-cysts, had 7 recoveries in 9 operations, and Boisiet, in 1867, read an essay, reproaching the timidity of French surgeons, who had so recoiled before it.—Thomas Dis. W., 727.

I shall not enter into any discussion of methods, as two are open to selection, viz., the abdominal and vaginal operation, and as these are fully referred to in works of reference, I shall not refer to it here. Dr. Mueller said, that while ovariotomy has been brought to a high degree ot perfection, the operation of hysterotomy has not yet passed the first stages of its development. Not only has the legitimacy of this operation been denied by experienced gynecologists, and the indications and contra-indications for its performance are yet declared to be unsettled. Nevertheless, so much has been done that it is claiming its appropriate place among the very grave surgical operations, and it is my conviction that men who study these questions are not justly chargeable with temerity or cruelty in offering such relief as may be found in this or such operations, being justified by the 29 per cent. of success obtained. Freund's operation is "*ad hoc sub judice.*"

